# INTEGRATING PRIMARY CARE AND COMMUNITY-BASED PROVIDERS IN AN INTERPROFESSIONAL TEAM FOR OLDER ADULTS

**DOI:** 10.1093/geroni/igad104.2604

**Published:** 2023-12-21

**Authors:** Denise Kropp, Danielle Clay, Susan Fosnight, Jennifer Drost, Michelle Hughes, Lorrie Warren, Jess Wilson

**Affiliations:** Northeast Ohio Medical University, Rootstown, Ohio, United States; Western Reserve Area Agency on Aging, Cleveland, Ohio, United States; Summa Health System, Akron, Ohio, United States; Summa Health Systems, Akron, Ohio, United States; Direction Home Akron Canton Area Agency on Aging & Disabilities, Uniontown, Ohio, United States; Summit County Probate Court, Akron, Ohio, United States; Community Health Care, Copley, Ohio, United States

## Abstract

The integration of healthcare and community-based providers has been long-recognized as the gold standard in the optimal care of older adults. Social determinants of health account for more than 80% of health outcomes. Therefore, optimal care requires meaningful collaboration between medical and community-based care providers. We present here a collaborative model of care integrating community providers and healthcare system. This is a long-standing collaborative approach where our Area Agency on Aging care managers present their complex cases to a team, which includes nursing, social work, geriatric medicine, and geriatric pharmacy. This team meets weekly for case presentations and interprofessional care planning. We have recently expanded these meetings to extend the reach of the team to remote Area Agencies on Aging, to ACO care managers, to a local FQHC, and to a local Habitat for Humanity affiliate for the Aging in Place initiative. Since September 2022 this expanded team reviewed 12 consumers. On average there were 4.25 medication issues identified (maximum=13, minimum=0). The team addressed the following in at least half of consumers: falls (58%), depression/anxiety (50%), advance care planning (67%), function (50%), caregiver support (58%), and dementia (50%). Additional issues included mobility (42%), finances (42%), pain, (33%), elder abuse (25%), safety (17%), behavioral health (17%), sleep (17%), spiritual care (17%), language barriers (17%), and substance abuse (8%). This team model has been shown to be replicable across provider settings. Expansion of this model extends the limited resources of geriatricians and geriatric pharmacists.

